# A social marketing perspective of young adults' concepts of eating for health: is it a question of morality?

**DOI:** 10.1186/s12966-020-00946-3

**Published:** 2020-03-30

**Authors:** Linda Brennan, Karen Klassen, Enqi Weng, Shinyi Chin, Annika Molenaar, Michael Reid, Helen Truby, Tracy A. McCaffrey

**Affiliations:** 1 School of Media and Communications, RMIT University, Building 9, 124 La Trobe Street, Melbourne, VIC 3004 Australia; 2 Department of Nutrition, Dietetics and Food, Monash University, Level 1, 264 Ferntree Gully Road, Notting Hill, VIC 3168 Australia; 3 Faculty of Arts and Education, Deakin University, 221 Burwood Hwy, Burwood, VIC 3125 Australia; 4 School of Economics, Finance and Marketing, RMIT University, Building 80, 445 Swanston Street, Melbourne, VIC 3000 Australia

**Keywords:** Young adults, Healthy eating, Social marketing, Behaviour change, Obesity, Behavioural typology, Segmentation, Marketing, Qualitative

## Abstract

**Background:**

Poor dietary choices are a risk factor for non-communicable diseases. Young adults have low levels of engagement towards their health and may not see the importance in the adoption of healthy eating behaviours at this stage in their lives. Here we utilise social marketing principles, digital ethnography and online conversations to gain insights into young adults’ attitudes and sentiments towards healthy eating.

**Methods:**

Young Australian adults who use social media at least twice a day were recruited by a commercial field house. Using a mixture of methods, combining online polls, forums and conversations, participants (*n* = 195, 18–24 years old) engaged in facilitated discussions over an extended 4 week period about health and eating-related topics. Data were analysed using thematic analysis constant comparison approach. A post-hoc conceptual framework related to religion was theorised and used as a metaphor to describe the results.

**Results:**

Findings demonstrate that different segments of young adults with varying attitudes and interest towards healthy eating exist. We developed a conceptual framework based on consumer segmentation which adopted religious metaphors as a typology of ‘consumers’. Some young adults practice and believe in the message of healthy eating (*saints*), whilst some oppose these messages and are not motivated to make any change (*sinners*), another segment are both aware of and interested in the issues but do not put healthy eating behaviours as a current priority (*person in the pew*).

**Conclusions:**

Consumer segmentation and social marketing techniques assist health professionals to understand their target audience and tailor specific messages to different segments. Segmentation provides insights on which groups may be most easily influenced to adopt the desired behaviours. The typology presented may be a useful tool for health professionals and social marketers to design strategies to engage young adults in healthy eating, particularly those *in the pew* who are contemplating a change but lacking the motivation. The utilisation of marketing segmentation in health promotion has the potential to enhance health messaging by tailoring messages to specific segments based on their needs, beliefs and intentions and therefore drive the efficient use of resources towards those most likely to change.

## Background

Poor dietary choices are a leading risk factor for non-communicable diseases (NCDs) and their associated deaths worldwide [1]. NCDs cause 70% of deaths globally, with higher mortality rates in more developed countries [2]. Improving the nutrition of populations is a key priority for many countries, evident through the global community’s commitment to achieving the Sustainable Development Goals (SDG) by 2030. Nutrition is at the core of many SDGs with 12 of the 17 relating to nutrition through their indicators [3]. Creating an environment that supports healthy choices and promotes the adoption of healthy eating habits is complex and requires input from many disciplines.

Healthy eating habits are becoming increasingly difficult to adopt in obesogenic environments, especially by young adults who have low levels of health engagement [4]. Previously identified barriers to healthy eating behaviours include lack of access to healthy foods, the expense of healthy food relative to unhealthy food, the social norm of unhealthy dietary patterns and lack of time and facilities to plan, shop, prepare and cook healthy foods and a general apathy of young adults towards their diet [5, 6]. Moreover, widespread marketing and promotion of non-core foods create cues that foster hunger, and influence preferences and override satiety signals leading to overconsumption of food energy on a regular basis [7]. In obesogenic environments, young adults need to be knowledgeable about any ‘issues’ and be able to adapt their behaviours to healthy alternatives if motivated to do so [8]. However, to be motivated and able to make such adjustments, they must first believe healthy eating is a viable alternative. Importantly, beyond the need for motivation there is the necessity that healthy options are available, accessible and affordable as well as desirable [9].

The behaviours, attitudes and beliefs across time, generations and cultural backgrounds are ever-changing. In today’s society we are more connected to information, technology, and social media [10] yet globally we are more overweight [11] and reported mental illness is on the rise [12]. Previous generations were connected to their community and culture in a face-to-face environment, with limited amounts of external input (i.e. advertising via newspapers and television). In the age of the internet, 89% of young adults now use social media platforms at least once daily [10]. This connection to a global audience has provided young adults with a platform to communicate with their peers as well as have greater exposure to advertising [13]. Both the food industry and lifestyle celebrities use a mix of engagement strategies on their social media pages with varying success [13] to attract consumers to their product. Strategies to engage followers can influence consumer attitudes [14] and consequently, their behaviour [15]. Health promotion organisations, however, have less of a presence and following on social media platforms and are not as successful in reaching their target audiences and delivering their messages [13].

Understanding how health professionals can create innovative and engaging social marketing strategies to motivate young adults to improve their health and provide strategies to overcome environmental influences that promote poor dietary choices, has the potential to improve their health status. Social marketing seeks to develop and integrate marketing concepts with other approaches to influence behaviours that benefit individuals and communities for the greater social good [16]. On the other hand, commercial marketing seeks to persuade people to buy, use or adopt products and /or ideas. It is successful because it is based on the premise that understanding your audience through market research will enable the marketer to more directly target the consumer’s needs and wants. Commercial marketing is all too successful when it comes to promoting the consumption of unhealthy food [17] and obesogenic environments are increasingly the norm [18].

Health professionals and social marketers both have a role to play in making healthy eating a desirable alternative for young adults. This paper aims to describe the findings of extended online conversations with young adults discussing health and food-related topics. Understanding their attitudes and beliefs towards healthy eating may help inform health professionals how to better engage with young adults via social marketing strategies. As this is a multi-disciplinary project, a glossary of terms that may be unfamiliar to some readers, is presented in Table 1. This glossary builds on terms presented in the study protocol paper ‘Communicating Health’ which describes the overall study in more detail [20].

**Table 1:** Glossary of terms

Term	Definition	In protocol
Challenge	A task presented to young adults by online moderators that requires thought and innovation to an issue or idea	No
Consumer segmentation	Market segmentation is the process of dividing a market of potential customers into groups, based on different characteristics. The segments created are composed of consumers who will respond similarly to marketing strategies and who share traits such as similar interests, needs, or locations. The idea behind segmentation is to create and resource different marketing strategies for different groups of consumers	Yes
Digital ethnography	Digital ethnography describes the process and methodology of doing ethnographic research in a digital space. The digital field site is sometimes comprised of text, video or images and may include social interactions	Yes
Journal entry	A log or diary kept by young adults to record their social media use and the content they were exposed to. This was a task within the online conversations that was separate to the forums	No
Lifestyle celebrity	A person who is famous or well known because of their perceived credibility and expertise in the health and lifestyle industry.	No
Living and Eating for Health Segment (LEHS)	These segments will be defined based on the outcomes of Phase 1 (online conversations and online survey) and evaluated throughout the project. Short descriptive segmentation personas will be developed to aid program development	Yes
Market research	Market research involves the process of gathering, analysing and interpreting information about people or companies (a market) to better understand their needs and preferences.	No
Online conversations	A multi-way dialogue between participants in an internet environment. It is informal, unstructured and dialogic (not mono-logic) in nature. It involves both listening and answering and develops over a period of time. It is not an online chat or interview	Yes
Online community	A virtual community where its members interact with each other around a shared interest, where interaction is mediated by technology via the internet. People join online communities through social networking sites, chat rooms, discussion boards, video games, blogs and virtual worlds.	No
Online forum	An online discussion group that allows its members with common interests to exchange open messages. It is hierarchical with a tree-like structure and may contain a number of sub-forums, each of which may have several topics.	No
Online panel	A group of research participants who have been selected to provide information in an online discussion forum at specified intervals over an extended period of time.	No
Segmentation “lens”	Analysing text from the online conversations using the healthy eating ‘segments’ developed through initial thematic analysis to help guide further analysis	No
Social marketing	Social marketing seeks to develop and integrate marketing concepts with other approaches to influence behaviours that benefit individuals and communities for the greater social good. It seeks to integrate research, best practice, theory, audience and partnership insight, to inform the delivery of competition sensitive and segmented social change programs that are effective, efficient, equitable and sustainable: Consensus definition International Social Marketing Association [19]	Yes
Social media platforms	Websites and applications that enable users to create and share content or to participate in social networking	Yes
Typology	A classification according to types or characteristics	No
Emerging Adulthood	A theory of a prolonged transition from adolescence to adulthood in people aged 18 to 25 years present in developed industrialised countries where adulthood has been delayed and is now occurring later in life than in previous generations	No
Social media influencer	A social media influencer is a person on social media who has established a large audience and credibility in a specific area e.g. fitness, nutrition, fashion	No

## Methods

This study reports findings from the formative phase (phase 1) of the ‘Communicating Health’ study using an online insights community methodology; the full protocol for phases one to four has been published elsewhere [20]. The online four-week long conversations aimed to provide formative information around young adult’s beliefs and attitudes towards healthy eating to inform subsequent phases of the ‘Communicating Health’ study. Different topics covered in the online conversations related to health and social media will be analysed in separate publications. Subsequent phases of the broader ‘Communicating Health’ study will provide quantitative and qualitative results to address the overall aims of the study related to health, healthy eating and social media use.

Using the principles of digital ethnography [21], online conversations involve the use of a powerful online insights community methodology (taking place in specifically-created virtual lounge rooms) and are used globally by major companies and health agencies to obtain insights into consumer behaviour [22]. Online conversations generate rich insights that go beyond the capabilities of traditional research approaches, as participants in the online communities’ converse over a longer period of time responding to questioning from group moderators.

### Recruitment procedure

Young adults (18–24 years old) using social media at least twice daily, living in Australia, were eligible to participate. The recruitment target was 200 young adults to provide rich data for understanding eating-related behaviours. This strategy was based on previous work using similar methodology [23]. Recruitment quotas were set to recruit a sample that was representative of the Australian population based on gender and location (percentages from different Australian States/Territories and Metropolitan/Regional locations) [24]. Young adults were recruited and online conversations were moderated over the four-week period by an Australian Market & Social Research Society-certified field house [25]. Those who had previously consented to participate in research across three research panels were invited to participate. Three panel partners were used to ensure a wide mix across the sample as well as to reach quotas. All panel partners were International Organization for Standardization (ISO) accredited by the Australian Market and Social Research Society to meet specific requirements for management and delivery of market and social research [26, 27]. Ethical approval for this study was provided by RMIT Business College Human Ethics Advisory Network; (Project number: 20489) and Monash University Human Research Ethics Committee (Project number: 7807).

The recruitment period began with the first participant registration on the 2nd May and continued until 1st June 2017 when the last participant registered. Panel members were emailed a screening survey by the field house, with seven questions on basic demographics, social media use and general health interest. Panel members, who met eligibility criteria, then within a week received an email invitation to complete the profiling survey and register. The survey contained 13 questions which took approximately 5 mins to complete and gathered data including self-reported weight and height, extent and frequency of social media use, as well as interest in health and demographics. At the end of this survey participants could register by creating an account on the virtual lounge rooms website which they were linked to at the end of the profiling survey. Participants were then assigned to one of four communities based on their age (18–21 years and 22–24 years) and interest in health. Interest in health classification was based upon the median value (Low being below median and mid-high being above median) for the following question: “On a scale of 1-7 where 1 means “Strongly Disagree” and 7 means “Strongly Agree”, please indicate how strongly you agree with the following statement – “I take an active interest in my health””.

### Recruitment challenges

**Fig. 1:**
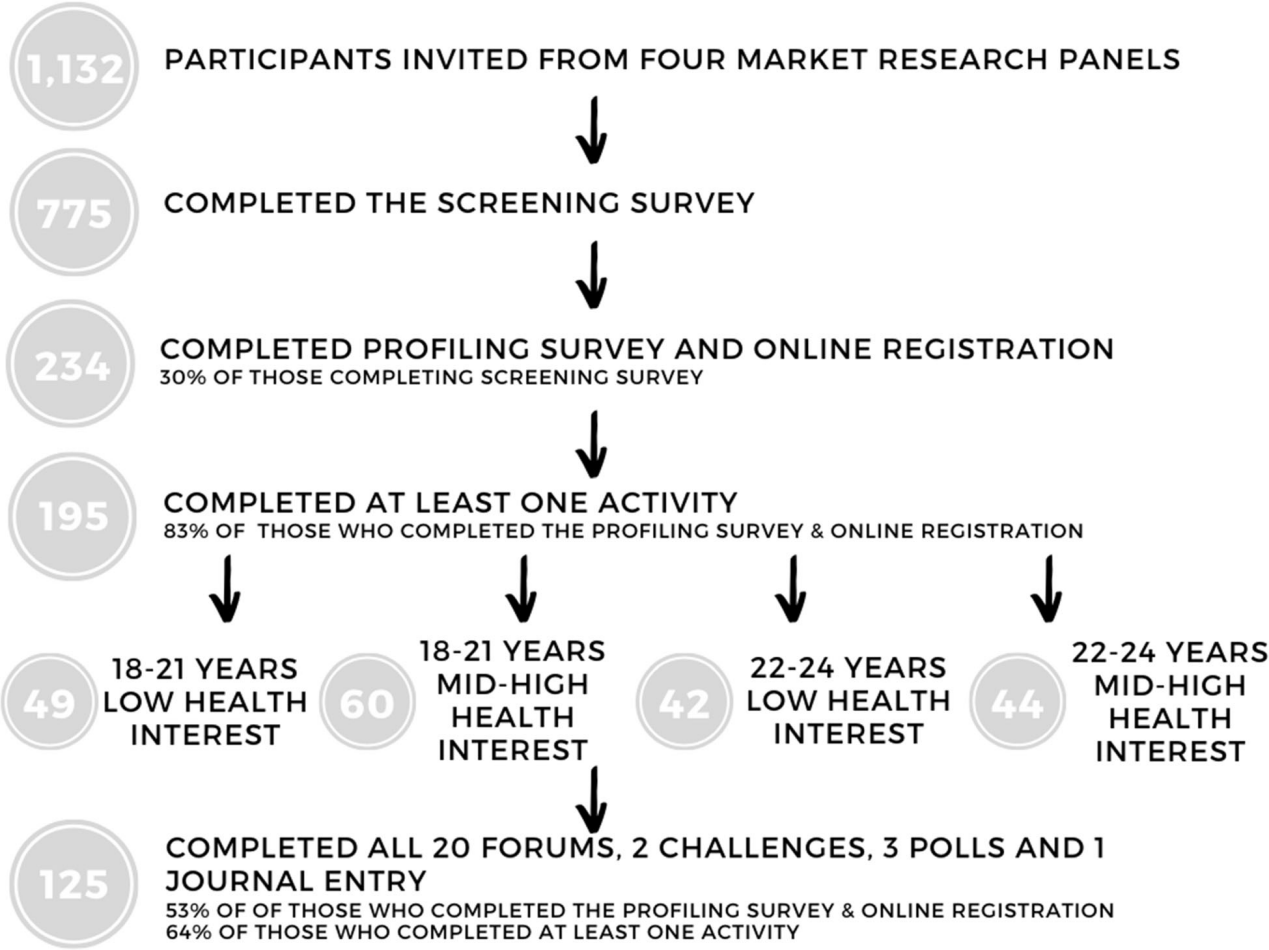
Study flow diagram

Young adults are notoriously difficult to engage with for the purpose of market research, especially for extended periods, and previous research has found similar difficulties in recruiting and retaining young adults [28, 29]. The drop-out rate was therefore large, which led the research team to increase targeting online panel members and implement a referral system whereby participants could invite their friends (who were also screened and profiled in the same way). The incentive for participating was an AU$100 gift voucher and twenty young adults with the most exhaustive contributions received an additional AU$100 (i.e. five adults per online community). Of the 775 young adults completing the screening survey, 234 registered for the online conversations and 195 participants engaged in at least one component of the online discussions (Fig. 1).

### Data collection

Data were collected over a four-week period; from 10 May 2017 to 6 June 2017. Once assigned to a community based on age and interest in health, each participant accessed the private online community which only researchers and participants had access to, by logging in and creating a username. A discussion guide was developed before the commencement of the study to provide an outline of topics and ideas to be explored [30]. The participants were asked to complete all 20 forums and two challenges which took approximately 5 mins each to complete (total of 110 min), one ongoing journal entry, which they were asked to complete at least four times and three polls. These were released on different days throughout the 4 week period however were kept open for participants to contribute to at their own time. Forums, challenges and polls were related to health, healthy eating and social media, and reflected on their experiences and observations. The conversations began with an introductory forum where moderators and participants introduced themselves in order to establish a relationship. Young adults also had the opportunity to respond to the insights of other participants and to prompts (e.g. social media adverts) guided by the online moderators from the market research field house. There were two moderators including a male (MMgt Marketing/Finance) and female (BA Psych Sociol, MA Applied Social Research) who both had extensive experience in market research. Data were in the form of text responses, uploaded images including memes and selfies to questions posed by moderators. Data saturation was apparent with later responses in the forums being indicative of previous responses.

### Data analysis

Online conversations were analysed in an exploratory hand-coding process on paper and using NVivo Version 11. As the analysis was exploratory, there was no a-priori concepts or models applied prior to commencement of data analysis. Researchers used thematic analysis and developed themes using a constant comparison approach [31]. Common themes associated with healthy eating behaviours and attitudes were used to form segments of people. Segmentation is a standard social marketing technique that goes beyond thematic analysis and grouping audiences based on demographics by creating groups of people based on their psychographics such as their attitudes, beliefs and behaviours [32]. During the analysis, young adults with similar dietary behaviours and attitudes towards eating were grouped together into segments. Additionally, comparative analysis was used on a continual basis to compare people and each group to check the segments were appropriately represented by the data. The moralisation of food and healthy eating were apparent throughout the initial analysis. A methodological approach in social marketing to explain the segments of people that were developed during coding, is to apply a post-hoc conceptual framework or typology of beliefs. In social marketing, using a conceptual framework promotes understanding of segmentation, as it provides an easily understood metaphor or common framework to elaborate on the segment’s beliefs, attitudes and behaviours. The identification of this conceptual framework, outlined in the results, was informed by the moralistic language used by young adults to describe food and health (e.g. ‘good’, ‘bad’, ‘guilt’). A post-hoc narrative literature search was conducted by LB and KK using the young adults’ language (e.g. ‘good’, ‘bad’, ‘guilt’) in the context of food and healthy eating. Religion was identified as a commonly occurring area relating to moralistic language that was similar to views expressed by participants throughout the online conversations. The research team then sought a researcher in the area of Religion (EW) who assisted in shaping the religious conceptual framework and narrative around the segments of people and their attitudes and beliefs related to healthy eating. This conceptual framework and the transtheoretical model of behaviour change [33] were used to describe all comments within the online discussions in the results and discussion.

Triangulation enhanced the transferability and dependability of research findings. This research used multiple forms of data (forums, online polls, challenges and a journal entry) in each aspect of the research in order to ascertain various perspectives on the issues. The data were analysed in duplicate by different members of the interdisciplinary team to ensure investigator triangulation [34]. This included two female researchers, a professor experienced in social marketing (LB) and a post-doctoral research fellow in the field of nutrition (KK). Where there were multiple potential interpretations of the data, consensus was reached via discussion amongst the team.

## Results

Participant characteristics are presented in Table 2. The mean age of participants was 21 years (SD ± 2), ranging from 18 to 24 years. The majority were female (61%), currently studying (70%) and living in a metropolitan area (80%). Disposable income varied between the participants; 39% reported spending less than AU$40/week, 30% reported having AU$40–$79/week and 31% reported having AU$80 or more/week. Less than half lived with their parents (42%), with the majority of young adults having alternative living arrangements including living with their partner, friends, housemates, alone, other family or with their children. More than a quarter (27%) spoke another language other than English at home or with their parents, with most culturally and linguistically diverse young adults reported having a Chinese or Indian cultural background.

**Table 2:** Descriptive characteristics of study participants (*n* = 195)

Characteristic	N (%) or Median (IQR)
**Age (years)**	
18–21 years old	109 (56%)
22–24 years old	86 (44%)
**Gender identity**^a^	
Female	119 (61%)
Male	75 (39%)
Non-binary/genderfluid/genderqueer	1 (1%)
**Body Mass Index (BMI) categories**^b^	*N* = 194
Underweight (BMI < 18.5)	16 (8%)
Healthy weight (BMI 18.5–24.9)	106 (55%)
Overweight (BMI 25.0–29.9)	42 (22%)
Obese (BMI ≥30.0)	30 (16%)
**Living location**	
Metro	156 (80%)
Regional/rural	39 (20%)
**Language other than English spoken at home/with parents**	
Yes	52 (27%)
No	143 (73%)
**Currently studying**	
Yes	137 (70%)
No	58 (30%)
**Level of current study**^**c**^	
High school, year 12	8 (6%)
TAFE, college or diploma	18 (13%)
University (undergraduate course)	97 (71%)
University (postgraduate course)	14 (10%)
**Highest level of completed education**^**d**^	
High school, year 10 or lower	2 (3%)
High school, year 11	2 (3%)
High school, year 12	13 (22%)
TAFE, college or diploma	23 (40%)
University (undergraduate degree)	16 (28%)
University (postgraduate degree)	2 (3%)
**Living arrangements**^**e**^	
Alone	24 (10%)
With their child (ren)	18 (8%)
With partner	37 (16%)
With other family	20 (9%)
With friend(s)/housemate(s)	34 (15%)
Living with parents	97 (42%)
I don’t wish to say	0 (0%)
**Dispensable weekly income**	
Less than AU$40	76 (39%)
AU$40–$79	59 (30%)
AU$80–$119	30 (15%)
AU$120–$199	17 (9%)
AU$200–$299	9 (5%)
AU$300 or over	3 (2%)
I don’t wish to say	1 (1%)
**Social media use frequency**	
More than twice a day	173 (89%)
Twice a day	22 (11%)
**Using social media to learn or talk about your health**	
Yes	128 (66%)
No	67 (34%)
**Interest in health**	
On a scale of 1–7, where 1 means “Strongly disagree” and 7 means “Strongly agree”, please indicate how strongly you agree with the following statement - I take an active interest in my health	6 (5, 6)

^a^ Based on the following question: “Please confirm your gender. Response options: Male; Female; Transmale/transman; Transfemale/transwoman; Non-binary/genderfluid/genderqueer; My gender is not listed (please specify)” [35]

^b^ BMI categories based on self-reported weight and height; one participant did not answer

^c^ Only participants currently studying answered this question

^d^ Only participants who were no longer studying answered this question

^e^ Participants could select more than one answer

A typology that was informed by religious metaphors, particularly from Christianity, was used to explain the initial analysis of young adult’s online discussions (Table 3). While we were not specifically looking for religious references or language throughout the analysis, it was apparent after a post-hoc literature search that the segments of people had beliefs around healthy eating that could be explained by the metaphor of religion. The metaphor of religion was chosen due to the strong connection with culture, food and eating. As the participant sample was primarily of Western culture, Christianity was used to shape the metaphor used as historically Western societies, including Australia, have been influenced particularly by Christianity. The metaphor was used to describe different segments of participants that had different beliefs in the ‘religion’ of healthy eating. The segments developed were *Saint*, *Sinner* and *Person in the Pew* with definitions outlined in Table 3. Throughout the forums, there was a discussion of morality around food choice:

*“So much of the way we look at food as pleasurable is about “indulgences” and “guilty pleasure” culture - “cheat days” or “i’m going to have to run this off later”. they all come with either guilt or shame or the idea of punishment after a satisfactory meal, as if food feeling good cant coexist with food being healthy. focusing on the feeling of having done something right rather than something wrong is really important.”***24 year old (yo) Non-binary/genderfluid/genderqueer**

**Table 3:** A conceptual framework for saints, sinners and persons in the pew in the context of food choices

	***Saint***	***Sinner***	***Person in the pew***
Definitions	1. a person acknowledged as holy or virtuous and regarded in Christian faith as being in heaven after death, a very virtuous, kind [36]. 2. In the Bible, the word “saints” refers to “holy people”. The people of Israel are “saints”, “holy ones”, a nation set apart by God for the worship and service of God, so in the New Testament those who comprise the church are also called holy, “saints”, because they too are set apart to God, God’s own people (Rom. 1.7; Phil. 1.1; passim) [37].	1. One who sins or does wrong; a transgressor [38]. 2. A scamp. a person who sins; transgressor [38]. 3. Noun 1. sinner - a person who sins, evildoer, offender, [38]. 4. .Sin can refer to a break in relations of humans with God or with other persons, an act that violates commandments and rules, or a power that tempts and dominates [39] 5. Sin defines the essence of sinners, so that they are their sin. Sinners commit acts of sin because they are essentially and totally defined by sin [39]	People who do believe in the issues, and have considered them and are sitting in the middle between saints and sinners.
Signifiers	• Adoption (of the message) • Self determined • Modelling ‘right’ behaviours	• Rejection • Fear • Transgression • Renunciation	• Avoidance • Guilt • Shame • Withhold/withdraw
Role	Actor/Exemplar	Rejector	Avoider
Motivation	Intrinsic	Amotivation	Extrinsic
Regulation (i.e. SDT)	Internal (self)	Non-regulation	External
Adapted definitions of people in the *“Western Church of Scientific Healthy Eating”*	People who are exemplars and set apart by their adherence to healthy eating *NOTE: researcher definition of healthy eating is according to prevailing scientific guidelines provided by organisations such as the World Health Organisation* [40]*, the Australian National Health and Medical Research Council* [41] *and the U.S. Department of Health and Human Services* [42]	People who oppose (actively or passively) the healthy eating messages they have heard (e.g. think the government is lying to them about obesity and healthy food).	People who accept the ideals of healthy eating but are not actively adopting these practices at present. Changing their eating habits is not a current priority. They think they are ‘good’.

The conceptual framework developed was used to highlight the different beliefs, attitudes and behaviours around food choice and healthy eating that different young adults practiced.

### Saints

*Saints* were described as those who are in agreement with the ideals and behaviours associated with healthy eating and were in the action or maintenance stages of behaviour change. They are exemplars for other young adults and are set apart by their adherence to healthy eating patterns. However, they are also characterised by balance: they can eat discretionary foods without feeling guilty, they may sin occasionally, but they make amends.

*“Soon after I watched the documentary forks over knives and I was shocked into action! I researched a plant-based diet for weeks and never looked back. I started to see health as something that starts from the inside. How can you feed a body crap with a side of sugar and expect it to last for 80 years? I see health as 80% diet and 20% exercise. now we eat an abundance of plants, grains, legumes, seeds and nuts and I have never felt healthier or stronger. I would like to implement more exercise. I have a 7 month old and any spare time is precious! I love talking about health and well-being. it's exciting to me because I have gone from not really caring to be so passionate about it!”***24yo Female (F)**

Saints promoted their views and healthy eating behaviours to others. They love sharing their ideas and propose new behaviours to others in their social group. They are self-determined and their rewards for their healthy eating behaviours are inherent, and therefore motivated to continue.

*“I associate health and well-being to being fit, able bodied, energetic, happy, social, peaceful, clear thinking, pride, being free of disease and illness and being supported. Some things that come to mind include exercise, green foods, whole foods, fruits, vegetables, friends, sunshine. I feel a sense of pride with myself hearing this term as I know I am doing the right things everyday to look about my overall health”***23yo F**

Then there were those who may have not known all that much about healthy eating but believed they did and were happy to say they were super healthy (and feel saintly as a result).

*“Well on other days for breakfast, I use my NutriBullet to make a big smoothie full of every green thing I can find in my fridge. For lunch next to our uni there is a restaurant that me an my friends usually go to. That place is super healthy and since we are regular customers, we often get discounts. For dinner I would usually get home and cook whatever. I'm off an asian heritage so a lot of our dishes are super healthy.”***23yo male (M)**

### Sinners

*Sinners* were those who opposed healthy eating ideals and rejected the message that obesity was a ‘real’ issue to their life and were most often in the pre-contemplation stage of behaviour change. They did not practice the ‘religion’ of healthy eating however, there were very few young adults who rejected the entire notion that healthy eating was important in some way. Sinners indicated they were content with their unhealthy lifestyle choices. In response to being shown advertisements supporting healthy eating messages’ sinners were happy with their unhealthy lifestyle choices:

*“The McDonald's ice cream catches my eye first [compared to the fruit smoothie advert shown] because I'm a bit of a fatty & love my bad stuff ! So I would definitely buy the ice cream with now seeing it makes me want one !!! Off to Maccas I go _ôÖ‰”***22yo F**

Only one individual outright rejected the concept of healthy eating:

*“I do not think about my health at all. If I see something I like, I eat it, without giving a damn. I also don’t exercise, because we are all gonna die anyway, so why not just enjoy life? I think I’ll gain weight over the next few years, but like I said, I don’t care.”***18yo M**

More often, *Sinners* felt some remorse eating unhealthy foods but the pleasure and enjoyment of eating those foods outweighed the guilt or pressure they felt to eat healthily so they continued eating the way they desired:

*“Well I always feel guilty when I take a cheat meal, since it makes me feel weak and bloated usually. But that feeling is destroyed by the amazing taste of pizza, so I guess it doesn't really trigger me.”***23yo M**

Others felt government-endorsed healthy eating messages were either false (“government is lying to me”) or over-exaggerated. In response to being shown a copy of the Australian Government’s Guide to Healthy Eating:

*“When I look at this image, based off what I see before, I just think, "urgh", in resentment, as I have seen this so many times and I think the standards set out are just outrageous for a 'healthy lifestyle'. It's so hard to incorporate these foods into your daily life - and it's not a necessity, like, you're not going to be an obese person with heart disease if you don't follow the rules of the diet.”***24yo M**

In response to a video advertisement about how fast food can contribute to central adiposity (increased fat around the waist):

*“It slightly gross and also an exaggeration on how bad junk food is. It is bad but not that bad on moderation and if you exercise. I like the positive message associated with it that it is promoting unhealthy people to stop eating junk food. The negative is that it feels like a bit of an over exaggeration. I don't think it is aimed at me because even though I eat junk food a lot , I cover it up by exercising a lot. I wouldn't share this with my friends because we would end up laughing at this ad and eating junk food anyway.”***19yo M**

*Sinners* focused on protecting themselves from ‘inconvenient truths’ by rejecting healthy eating messages while at times adopting other ‘healthy’ messages such as those related to exercise. They were content with their current ‘religion’ or way of eating, so would be unlikely to be easily persuaded to change and were often not in any way on the path to making a change. Thus, extant social marketing strategies are insufficient for *sinners* to alter their dietary behaviours or beliefs.

### Person in the pew

The *person in the pew* was characterised by espoused beliefs in the ideals of healthy eating but an overall lack of ‘active engagement’ with the actions and behaviours required to perform healthy eating within their lifestyles. Many individuals were in the contemplation stage of behaviour change in relation to healthy eating, but it was currently not a priority for them. They did not plan to make any immediate changes to their diet, although they may consider doing so in the future. They may be weighing up the pros and cons of changing their habits, but the pros are not strong enough to require immediate action. They may be interested in health and healthy eating, and may even actively seek or be passively exposed to health information online or on social media platforms. They sometimes/often had healthy eating knowledge and beliefs, and felt like they should be eating healthier, which led to feeling guilty. Guilt did not appear to improve eating habits. The language used to describe foods as ‘good’ and bad’ reflected the moralisation of food by people in this category. They were often ‘tempted’ by ‘bad’ foods and gave in to their temptations.

*“I try to be healthy but usually the temptation of tasting new and good food is too much!”***18yo F**

There were also those who acknowledged they were NOT *sinners* but were honest about their struggles:

*“I constantly think about my health: i try to change but in the end i jst [sic] don't have the will power too, which really sucks and my life is so busy i cant work out so eating healthy is my only option and i don't even do that very well.”***22yo M**

and

*“Whenever I hear those terms [healthy and wellbeing] being thrown around I feel happy when I hear these terms because I know I do it majority of the time but when I'm eating shit I feel crap when I hear it.”***19yo F**

While they may be feeling guilty – “I don’t want to do that, even though I know it’s healthy”, they found it difficult to do the right thing most of the time and hated the sense of being judged.

*“Don't judge but my guilty pleasure is KFC! i love nothing more than a zinger combo meal with wicked wings and to make myself feel better i substitute the potato and gravy for coleslaw (so much healthier... note [sic]).”***24yo F**

and

*“When I hear the term 'being healthy' I kind of feel a bit guilty because I know I don't eat healthily or treat my body as a temple. I smoke cigarettes, and though I've been cutting down, it's been a big thing for a while. I would love to quit smoking and get back into eating healthy and wholesome foods that give me the feeling of clean achievement and gratification.”***21yo F**

There were also those who were looking for signs of reassurance from others that they could be ‘good’ too. For example,

*“I like seeing real people who comment and post to show that what theyve been told is in fact working! I found it really interesting though, how many people also struggling with maintaining healthy meal schedules too! I for one, will eaither not eat at all through the day from being too busy, and then will splurge at night, or Ill eat all the wrong foods!”***18yo F**

And those who faced temptation in their own way:

*“My first impression are the smoothie looks really good but I not sure if I would try it as I can't stand avocado where as the the ice cream I try to look away from cause I love ice cream but I know it's so wrong for me”***23yo F**

For the *person in the pew*, the pros of healthy eating were all the positives associated with feeling healthy (i.e. worthy and ‘good’). However, the pros were often outweighed by the cons and were insufficient to make them prioritise healthy eating. The cons included time, taste, money, lack of capacity to engage, difficulty and effort to cook healthy food.

*“I always struggle in eating healthy since I'm incredibly busy.”***23yo M**

and

*“The first thing that comes to mind with the term "healthy" is confronting magazine cover about fad diets or television ads about losing weight. Therefore it has started to make me feel indifferent about "well-being" because of all the societal pressure of what health looks like. I do occasionally think about my health, but it isn't really my priority right now, as often university takes priority over other aspects of my life. I think my health-consciousness will change over the next few years, as university work will get harder and I will find that I need to put more effort into giving myself the energy to balance many aspects of my life.”***18yo F**

Most supported the healthy eating notion but their beliefs were not strong enough to motivate them to take action. They relied on external motivations such as those described above and let others take the lead in healthy eating behaviours. Overall, the *person in the pew* segment understood the message and accepted that the messenger had their benefit in mind.

## Discussion

In this formative research, we developed young adults segments that were along the continuum of the transtheoretical model of behaviour change [33] from pre-contemplation (*sinners*), contemplation and preparation (*people in the pew*) to action and maintenance (*saints*) overlaid with a typology of Christianity. We found many young adults in our study were contemplating healthy eating, but this segment, the “*people in the pew*”, were struggling to make this a priority. Others were actively leading a healthy life through their eating-related behaviours (*saints*). We also found a proportion of young adults who were resistant to the healthy eating messages and or had no interest or motivation to alter their current dietary habits (*sinners*).

Eating healthily when everything seemingly conspires against healthy choices requires a degree of motivation many young adults seem not to be able to achieve long term [43]. The perceived lack of short-term tangible outcomes from healthy eating may contribute to low motivation in this age group, alongside the prioritisation of other aspects of life such as study and mental wellbeing (Molenaar, A, Choi T, Brennan L, Reid M, Lim M, Truby H et al. Language of health of young adults: Qualitative exploration of perceptions of health, wellbeing and health promotion via online conversations, submitted). First proposed by Deci and Ryan [44] the Self Determination Theory (SDT) posits people can be self-motivated and self-regulating in terms of their own behaviours and motivation and rewards can be self-generated or intrinsic. The self-determination continuum suggests intrinsic self-motivated behavioural regulation is at the ‘upper’ end of the behavioural spectrum (self-determined). Young adults who were recognised as *saints* sat at this end of the continuum (see Table 3). At the opposite end is the state of ‘amotivation’. A person in this state is not motivated at all and does not ‘intend’ to behave in any way, except as they choose. These are the *sinners*. This may be due to a lack of self-efficacy [45] or a lack of interest. Thus, on the continuum, they are considered as non-regulated and non-self-determined. However, the meaning of non-self-determined in this model may be normative – that someone else thinks you should do something does not mean you think you should do something. Hence, ‘amotivation’ is not necessarily a state that can be changed easily with some externally applied incentives to alter behaviour in a certain way.

### Theorising saints and sinners

Behaviours and practices related to food have historically had a strong connection with religions. Practices of abstinence (e.g. fasting during Lent or Ramadan), restriction (e.g. no meat on a Friday) and avoidance (e.g. Hindus do not eat beef because the cow is viewed as a sacred animal) are reflected across different religions [46]. Food, or the absence of food, acts as a conduit between the physical and spiritual world through which one could attain otherworldly transcendence [47].

The Christian meta-narrative has a historically strong influence on Western societies, shaping core principles, attitudes and values that are translated into everyday practices [48]. While Christian influence has been on a decline and Western societies are increasingly non-religious and secular in their outlook of life, the location of the sacred has also shifted from the religious towards secular activities in religion-like ways [49–52]. Moralism, which is closely associated with Christianity, has become dislocated and decentred from religious institutions and applied across secular understandings [52]. Likewise, religious food practices have shifted; the Catholic practice of eating fish on Fridays has evolved towards eating more energy-dense, convenience foods as a result of modern time-poor ‘life’. Fasting, formerly a part of religious tradition has also become the core aspect of trendy intermittent fasting 5:2 and 16:8 diets to manage overall health and well-being as well as lose weight. Similarly, religious expressions such as “temptation”, “heavenly” and “sinfully delicious” have been used by the fast food industry to describe food without direct association with their religious meanings [53].

As religious attitudes and behaviour become more implicit, the Christian moralising of ‘good’ and ‘evil’ has become part of the culture of food and dieting too. This discourse of purity and profanity can be located in food practices concerning abstinence, restriction and avoidance [54]. The moralisation of food consumption is most evident, for example, in discourses of guilt in food choices [55, 56] and the focus on specific dietary trends [57, 58].

For example, orthorexia, defined as a ‘healthy anorexia’, has been viewed as an exercise of purification particularly in young females, where they limit their food intake to an amount beneath the national dietary recommendations [59]. Participants in this Australian study emphasised dietary preference as a way to differentiate themselves from others [59]. This voluntary povertisation of the body has a ‘long association with relinquishing the worldly and embracing the spiritual’ [60]. Moral superiority over choices of food, whether in consumption and abstinence, has characteristics similar to the spiritual pursuit of purity and holiness.

The conceptual framework presented in Table 3 draws on definitions from Christianity, as Western culture remains predominantly influenced by this religion. Although Christianity and religion can be contested terms that are subjective in practice [61], some level of religious literacy and their cultural applications were evident in Australian media and popular discourses [52]. As such, we propose healthy eating has religion-like properties as a system of beliefs that can be similarly pursued with great interest [61, 62]. When it comes to healthy eating though, being a *sinner* can be perceived as being much easier than being a *saint* when everything is presented to you as a sacrifice: fun, taste, convenience, cost, effort and time have to be surrendered in order to be healthy. It all appears to be too hard (unless you are a *saint*). Evidently, it cannot be assumed everyone is waiting for the ‘light’ of how to incorporate healthy beahviours into their lives. Some young adults are quite comfortable with their current state of health and well-being; they simply do not care about the issue of healthy eating and are unlikely to change their behaviour.

Often the literature ‘assumes’ all individuals are prone to feeling food guilt [55, 63], however, it seems not everyone feels guilty when they eat ‘naughty’ foods [56, 64]. A qualitative study exploring the eating behaviours of Canadian teenagers living in both urban and rural areas found a small proportion had no feelings of guilt or regret when they consumed fast food [56]. These teens recognised fast food was unhealthy yet, similar to the young adults classified as ‘*sinners*’ through this research, participants enjoyed eating these foods without any concern for their health [56]. This may explain why messages of fear, guilt and shame do not work to change the behaviours of some individuals [65].

Rejecters of healthy eating messages are not only apparent in young populations; older adults can also be sceptical of the ‘healthist ideology’ [64]. Delaney et al. [64] explored the moral perceptions of food in older Irish adults and found some did not believe in the messages around healthy eating. Those that rejected the messages (and did not subscribe to the healthy eating ideology) were sceptical of the constant conflicting nutrition advice and questioned the motives of those providing information. Others justified poor dietary habits by adopting a fatalism perspective and based their views on anecdotal evidence that good health is not always an outcome of lifestyle choices such as a healthy diet.

When it comes to behaviour change it is important to note that while fear, guilt and shame appeals are widely used, guilt appeals do not work on many young adults who do not hold the same beliefs (e.g. *sinners*). Guilt and shame are socially-constructed emotions, thus, guilt appeals only work on people who are connected to the issue in some way. Such appeals are only useful if the person believes in and cares about the idea, such as the ‘*person in the pew*’ and the ‘*saint*’. New communication techniques are needed to engage young adults, namely ‘*sinners*’, who do not respond to messages aiming to evoke negative feelings around food. As such, our next steps will be to move away from fear, guilt and shame based messaging that religious metaphors may imply.

The findings from this study will inform the development of a survey that will be used in the next stage of the ‘Communicating Health study’ [20]. This online survey will help evaluate and further define the segments that were developed from the online conversations by recruiting a larger sample of young adults [20]. Subsequent phases (two to four) of the Communicating Health study will involve co-designing social marketing campaigns with young adults including those aimed at specific segments. This will allow for greater understanding on how to tailor messaging and health campaigns towards specific segments of young adults. A key strength of this study is the rich understanding gathered from the online conversations about young adults’ attitudes, beliefs and behaviours towards healthy eating. Additionally, the developed segments (*saint*, *sinner* and *person in the pew*) have provided valuable insight into the variations young adults’ have to the uptake and acceptability of healthy eating messages through social marketing. However, several limitations in this study are acknowledged. The majority of young adults who participated are not necessarily representative of the Australian population. Data collection took place during the Australian university exam period, which may have contributed to the challenges associated with recruitment and attrition. The post-hoc application of the religious metaphor was not confirmed with the young adults, due to the study design. However, applying metaphors is a common method within the field of social marketing to allow a wider understanding of the group.

## Conclusions

The majority of participants in our study appeared to believe in the ideals of healthy eating; either as *saints* or *people in the pew*. Most young adults had an interest in health and healthy eating, but many were struggling with actually “living” it. This interest in health bodes well for health professionals and social marketers aiming to establish healthy eating patterns, however the struggle speaks to the necessity for aligned environmental shifts that make healthy eating more viable. There were also those, however, who were disinterested in or actively opposed the idea of healthy eating; the *sinners*. Understanding how to best engage and motivate this group of resistant young adults will be essential when developing strategies addressing the notion of healthy eating to those who oppose it.

This paper contributes to a more nuanced understanding of human behaviours in a very complex system. While segmenting young adults using religious metaphors is fraught with connotations, the fight over ‘good’ and ‘bad’ food has attributes of religious fervour. Typecasting food as the enemy is unlikely to attract adherents to the cause of healthy eating. Food is a fundamental human need and the abundance of energy-dense food is a modern phenomenon of which we have limited understanding of the potential management of its negative outcomes. The typology presented may be a useful tool for health professionals and social marketers to design strategies to include the range of young adults engaged in healthy eating, particularly those *in the pew* who are contemplating a change but lacking the motivation, and *sinners* who have no motivation at all to adopt healthy eating behaviours. The utilisation of marketing segmentation in health promotion has the potential to enhance health messaging by tailoring messages to specific segments based on their needs, beliefs and intentions and therefore drive the efficient use of resources.

This research shows the importance of understanding the various beliefs young adults have around healthy eating. Further research is required to understand how to keep the *person in the pew*, in the pew, and how they can gain the motivation to make healthy eating a priority. Health professionals seeking to address obesity through social marketing strategies need ideas and tools that are implementable by young adults in their normal daily lives. Strategies must engage and resonate with young adults and be tailored to each perspective (*saint*, *sinner* or *person in the pew*) depending on where their beliefs lie in order to be effective.

## Supplementary information

**Additional file 1.** Sample of the study.

## Data Availability

The datasets generated and/or analysed during the current study are not publicly available as consent was not provided by the participants to provide their responses outside of the study team but are available from the corresponding author on reasonable request.

## Abbreviations

F: Female
M: Male
NCDs: Non-communicable diseases
SDG: Sustainable Development Goals
SDT: Self Determination Theory

## References

[1] Emmanuela G, Ashkan A, Amanuel AA, Kalkidan HA, Cristiana A, Kaja MA (2017). Global, regional, and national incidence, prevalence, and years lived with disability for 328 diseases and injuries for 195 countries, 1990-2016: a systematic analysis for the global burden of disease study 2016. Lancet.

[2] World Health Organization. The top 10 causes of death. 2018. https://www.who.int/news-room/fact-sheets/detail/the-top-10-causes-of-death. Accessed 12 Dec 2018.

[3] International Food Policy Research Institute (2016). Global nutrition report 2016: from promise to impact: ending malnutrition by 2030.

[4] Goddings A-L, James DR, Hargreaves DS (2012). Distinct patterns of health engagement in adolescents and young adults: implications for health services. Lancet.

[5] Munt AE, Partridge SR, Allman-Farinelli M (2017). The barriers and enablers of healthy eating among young adults: a missing piece of the obesity puzzle: a scoping review. Obes Rev.

[6] McGowan L, Caraher M, Raats M, Lavelle F, Hollywood L, McDowell D (2017). Domestic cooking and food skills: a review. Crit Rev Food Sci Nutr.

[7] Cohen DA (2008). Obesity and the built environment: changes in environmental cues cause energy imbalances. Int J Obes.

[8] Parkinson J, Schuster L, Russell-Bennett R (2016). Insights into the complexity of behaviours: the MOAB framework. J Soc Mark.

[9] Palermo C, McCartan J, Kleve S, Sinha K, Shiell A (2016). A longitudinal study of the cost of food in Victoria influenced by geography and nutritional quality. Aust N Z J Public Health.

[10] Sensis (2017). Sensis Social Media Report 2017: Chapter 1 - Australians and social media.

[11] Organisation for Economic Co-operation and Development. Obesity Update 2017. OCED. 2017. https://www.oecd.org/health/health-systems/Obesity-Update-2017.pdf. Accessed 12 Dec 2018.

[12] James SL, Abate D, Abate KH, Abay SM, Abbafati C, Abbasi N (2018). Global, regional, and national incidence, prevalence, and years lived with disability for 354 diseases and injuries for 195 countries and territories, 1990–2017: a systematic analysis for the global burden of disease study 2017. Lancet.

[13] Klassen K, Borleis E, Brennan L, Reid M, McCaffrey TA, Lim M (2018). What people "like": analysis of social media strategies used by food industry brands, lifestyle brands, and health promotion organizations on Facebook and Instagram. J Med Internet Res.

[14] Buchanan L, Kelly B, Yeatman H (2017). Exposure to digital marketing enhances young adults’ interest in energy drinks: an exploratory investigation. PLoS One.

[15] Fishbein M (2000). The role of theory in HIV prevention. AIDS Care.

[16] Brennan L, Binney W, Parker L, Aleti T, Nguyen D (2014). Social marketing and behaviour change: models, theory and applications.

[17] Aschemann-Witzel J, Perez-Cueto FJ, Niedzwiedzka B, Verbeke W, Bech-Larsen T (2012). Lessons for public health campaigns from analysing commercial food marketing success factors: a case study. BMC Public Health.

[18] Hughey SM, Kaczynski AT, Porter DE, Hibbert J, Turner-McGrievy G, Liu J (2019). Development and testing of a multicomponent obesogenic built environment measure for youth using kernel density estimations. Health Place.

[19] Australian Association of Social Marketing: What is social marketing? 2016. https://www.aasm.org.au/what-is-social-marketing/. Accessed 2 May 2019.

[20] Lombard C, Brennan L, Reid M, Klassen KM, Palermo C, Walker T (2018). Communicating health-Optimising young adults’ engagement with health messages using social media: study protocol. Nutr Diet.

[21] Brennan L, Fry M-L, Previte J (2015). Strengthening social marketing research: harnessing “insight” through ethnography. Australas Mark J.

[22] Dessart L, Veloutsou C, Morgan-Thomas A (2015). Consumer engagement in online brand communities: a social media perspective. J Prod Brand Manage.

[23] VicHealth (2013). Drinking-related lifestyles: exploring the role of alcohol in Victorians’ lives.

[24] Australian Bureau of Statistics (2016). 3101.0 - Australian Demographic Statistics, Jun 2016.

[25] Australian Market and Social Research Society (2018). Code of Professional Behaviour.

[26] International Organization for Standardization (2018). The facts about certification.

[27] Australian Market and Social Research Society (2019). ISO Standard for market social and opinion research.

[28] James A, Taylor B, Francis K (2014). Researching with young people as participants: issues in recruitment. Contemp Nurse.

[29] Leonard A, Hutchesson M, Patterson A, Chalmers K, Collins C (2014). Recruitment and retention of young women into nutrition research studies: practical considerations. Trials.

[30] Klassen K, Reid M, Brennan L, McCaffrey TA, Truby H, Lim M (2017). Methods - Phase 1a Online Conversations screening and profiling surveys and discussion guide.

[31] Boeije H (2002). A purposeful approach to the constant comparative method in the analysis of qualitative interviews. Qual Quant.

[32] Slater MD (1996). Theory and method in health audience segmentation. J Health Commun.

[33] Prochaska JO, Velicer WF (1997). The transtheoretical model of health behavior change. Am J Health Promot.

[34] Patton MQ. Qualitative research and evaluation methods. Thousand Oaks: SAGE; 2002.

[35] Australian Bureau of Statistics. 1200.0.55.012 - Standard for Sex and Gender Variables, 2016. https://www.abs.gov.au/ausstats/abs@.nsf/latestProducts/1200.0.55.012Media%20Release12016. Accessed 20 Apr 2017.

[36] Oxford University Press (2019). Oxford English Dictionary.

[37] Livingstone EA. The concise Oxford dictionary of the Christian Church. 3rd ed. New York, NY: Oxford University Press; 2013.

[38] The Free Dictionary (2019). The Free Dictionary by Farlex.

[39] McCurley FR, Hübner H, Schmiechen P, DeYoung RK (2011). Sin: Encyclopedia of Christianity Online.

[40] World Health Organization, Food and Agriculture Organization of the United Nations. Driving commitment for nutrition within the UN Decade of Action on Nutrition: World Health Organization; 2018. https://apps.who.int/iris/bitstream/handle/10665/274375/WHO-NMH-NHD-17.11-eng.pdf?ua=1. Accessed 30 Jul 2019.

[41] National Health and Medical Research Council. Australian Dietary Guidelines: National Health and Medical Research Council; 2013. https://www.eatforhealth.gov.au/sites/default/files/content/n55_australian_dietary_guidelines.pdf. Accessed 30 Jul 2019.

[42] U.S. Department of Health and Human Services and U.S. Department of Agriculture (2015). 2015–2020 Dietary Guidelines for Americans.

[43] Michie S, Ashford S, Sniehotta FF, Dombrowski SU, Bishop A, French DP (2011). A refined taxonomy of behaviour change techniques to help people change their physical activity and healthy eating behaviours: the CALO-RE taxonomy. Psychol Health.

[44] Deci E, Ryan R. Intrinsic motivation and self-determination theory. New York: Plenum; 1985.

[45] Deci EL, Ryan RM (2002). Handbook of self-determination research.

[46] Harvey G (2014). Food, sex and strangers: understanding religion as everyday life.

[47] Hamilton M (2000). Eating ethically:'spiritual' and 'quasi-religious' aspects of vegetarianism. J Contemp Relig.

[48] Woods T (2012). How the catholic church built western civilization.

[49] Demerath I, Nicholas J (2000). The varieties of sacred experience: finding the sacred in a secular grove. J Sci Study Relig.

[50] Knott K, Day A, Vincett G, Cotter CR (2016). The secular sacred: in between or both/and?. Social identities between the sacred and the secular.

[51] Lynch G (2012). The sacred in the modern world: a cultural sociological approach.

[52] Weng E (2020). Media perceptions of religious changes in Australia: of dominance and diversity.

[53] Contois EJ (2015). Guilt-free and sinfully delicious: a contemporary theology of weight loss dieting. Fat Stud.

[54] Douglas M (1980). Purity and danger: an analysis of concepts of pollution and taboo.

[55] Bui MM, Tangari AH, Haws KL (2017). Can health “halos” extend to food packaging? An investigation into food healthfulness perceptions and serving sizes on consumption decisions. J Bus Res.

[56] McPhail D, Chapman GE, Beagan BL (2011). "too much of that stuff can’t be good": Canadian teens, morality, and fast food consumption. Soc Sci Med.

[57] McCartney M. Margaret McCartney: clean eating and the cult of healthism. BMJ. 2016;354:i4095. 10.1136/bmj.i4095 27456087

[58] Knight C. “An alliance with mother nature” : natural food, health, and morality in low-carbohydrate diet books. Food and Foodways. 2012;20(2):102–22.

[59] Musolino C, Warin M, Wade T, Gilchrist P (2015). ‘Healthy anorexia’: the complexity of care in disordered eating. Soc Sci Med.

[60] Alderton Z. 'As Holy as Serving the Homeless': Orthorexia as a Modern Religious Pursuit. Australian Association for the Study of Religion and New Zealand Association for the Study of Religion Annual Conference; 8 December; University of Notre Dame 2017.

[61] Smith JZ, Taylor MC (1998). Religion, religions, religious. Critical terms for religious studies.

[62] Boyan SA (1967). Defining religion in operational and institutional terms. Univ Pa Law Rev.

[63] Geyskens K, Dewitte S, Pandelaere M, Warlop LUK (2008). Tempt me just a little bit more: the effect of prior food temptation Actionability on goal activation and consumption. J Consum Res.

[64] Delaney M, McCarthy MB (2014). Saints, sinners and non-believers: the moral space of food. A qualitative exploration of beliefs and perspectives on healthy eating of Irish adults aged 50-70. Appetite.

[65] Brennan L, Binney W (2010). Fear, guilt and shame appeals in social marketing. J Bus Res.

