# Supplementary material for: A social marketing perspective of young adults' concepts of eating for health: is it a question of morality?
Source: Int J Behav Nutr Phys Act. 2020 Mar 30;17:44. doi: 10.1186/s12966-020-00946-3 (PMC7106857; doi:10.1186/s12966-020-00946-3)
Supplement: Supplementary file 1 — Additional file 1. Sample of the study. [file 12966_2020_946_MOESM1_ESM.docx]

**Sample of the study**

This study aimed to recruit a population of Australian young adults (aged 18-24 years old) with no restriction on BMI, socio-economic status or ethnicity. The sample was recruited by a market research field house using standard procedures from their pre-existing panels of participants within their database. Participants from panels were eligible if they were aged between 18-24 years old, currently residing in Australia and self-reported using social media at least twice a day. These participants were then allocated to one of four groups intended to have approximately even numbers based on age; 18-21 or 22-24, and interest in health; low or mid/high.

A total of 775 people completed the screening questionnaire and of those 234 that registered for the online conversations, 195 participants completed at least one of the forums/challenges within the online conversations analysed in this manuscript. The sample of participants completing the forums/challenges analysed in this manuscript were both male and female, despite there being slightly more females (61%). There were slightly more participants aged 18-21 years (56%) than 22-24 year olds (44%). There were a range of people within BMI categories and other socio-economic variables and the majority were students (70%).
